# Potential efficacy of existing drug molecules against severe fever with thrombocytopenia syndrome virus: an in silico study

**DOI:** 10.1038/s41598-021-00294-7

**Published:** 2021-10-21

**Authors:** Shilpa Chatterjee, Choon-Mee Kim, Dong-Min Kim

**Affiliations:** 1 Department of Biomedical Science, College of Medicine, Chosun University, Gwangju, Republic of Korea; 2 Premedical Science, College of Medicine, Chosun University, Gwangju, Republic of Korea; 3 Department of Internal Medicine, College of Medicine, Chosun University, 588 Seosuk-dong, Dong-gu, Gwangju, 501-717 Republic of Korea

**Keywords:** Computational biology and bioinformatics, Virtual drug screening

## Abstract

Severe fever with thrombocytopenia syndrome (SFTS) is a zoonotic disease caused by the SFTS virus (SFTSV). SFTS can be considered a life-threatening notifiable infectious disease. The unavailability of specific therapeutics encourages the investigation of potential efficacy of existing drugs against this infection. Drug repurposing was done by performing  virtual screening of already established drug molecules followed by 100 ns molecular dynamics simulations and molecular mechanics Poisson–Boltzmann surface area–based binding-energy calculation by targeting the SFTS L protein. On the basis of binding energy and protein–ligand interactions, top 10 promising hits were identified, showing stable binding with SFTS L protein. Further 100 ns atomistic MD simulation refined the hits from top 10 to top 4 with docking-based binding energy lesser than −8.0 kcal/mol toward the SFTS L protein and engaged in π–π interactions with pivotal amino acid residues. Various parameters and binding affinity of top 4 ligands towards L protein was computed. Ligand zaltoprofen exhibited best binding energy −220.095 kJ/mol. The present work is the first in silico study to assess bromfenac, cinchophen, elliptinium, and zaltoprofen; four promising hits against SFTS. Nonetheless, further proper biological evaluation is necessary to determine their efficacy against SFTS.

## Introduction

Severe fever with thrombocytopenia syndrome (SFTS) is a zoonotic disease caused by the SFTS virus (SFTSV), genus Phlebovirus, family Bunyaviridae. The first case of SFTS was identified in Huaiyangshan in the Henan province of China in 2011, with major clinical symptoms of severe fever and thrombocytopenia^1^⁠. Later, in 2012, such infection cases were identified in Korea, where a female patient was infected by a tick and died of multiple organ failure^2^⁠. *Haemaphysalis longicornis* ticks widespread in the Republic of Korea are believed to be a vector of SFTSV^3^⁠. According to some studies, the case fatality ratio of SFTS is 6.3–30%, meaning a high mortality rate, which primarily affects China, South Korea, and Japan^4,5^⁠. Along with tick-borne transmission, a case of non-vector SFTSV transmission has also been reported^4^. Although SFTS can occur throughout the year, the high-risk tick bite season is between spring and autumn, with a high mortality rate. Despite its clinical and public health importance, currently, no safe and effective pharmacological and vaccine options are available for SFTS; this situation urgently requires the development of quality treatment options for this potentially severe disease. Although, ribavirin has been reported to be effective against SFTSV but failed to modify the disease outcome in patients with low viremia^5^. Some research groups have also reported the effectiveness of favipiravir at treating SFTS in both animal models and humans, but a human trial with a limited sample size failed to show treatment efficacy sufficient for mass use^6,7^⁠. Thorough investigation of more effective and targeted drugs against SFTSV is needed to prevent the associated deaths.

SFTSV is a single-stranded enveloped RNA virus that has a tri-segmented genome; namely, the genome consists of a large (L) segment, medium (M) segment, and small (S) segment^5^. The L segment is the major contributor to virus transcription and encodes viral RNA-dependent RNA polymerase with a size between 250 and 450 kDa^6^⁠. The M segment encodes the envelope glycoprotein, which facilitates host cell entry and mediates virion maturation and assembly. The S segment, which is a small genome part, encodes a nucleoprotein and a nonstructural protein (Nss)^7^⁠. The L segment is the pivotal part of the genome of bunyaviruses. This part of the genome encodes 3 distinct RNA species, including antigenomic complementary RNA, genomic viral RNA, and a capped, mostly nonpolyadenylated viral mRNA that contributes to viral replication and transcription. The mechanism behind SFTSV genome replication is believed to be a de novo process, but the transcription of SFTSV genes is solely dependent on the phenomenon of cap-snatching, where cellular RNAs are involved^8^⁠. Targeting the L segment is believed to suppress SFTSV replication by inhibiting viral transcription and replication^5^. Hence, the multifunctional and multidomain characteristics of the L segment of bunyaviruses make it an ideal drug target in SFTSV. Vogel et al. (2020) reported the structure of the cap-binding domain of the SFTSV L protein complexed with a triphosphate inhibitor: 7-methyl-guanosine-5′-triphosphate (MGP)^6^.

It is a well-established fact that drug discovery is expensive and time consuming. Therefore, considering the urgency of finding effective drug candidates against an emerging disease such as SFTS, we decided to perform a drug-repurposing study by screening a database of FDA-approved small-molecule. Drug repurposing is an approach to drug development whereby we can reuse triphosphate like drugs already proven to be effective against other diseases^9^⁠. This approach is becoming an efficient, cost-effective, and universal strategy with numerous advantages. In the present study, structure-based virtual screening of the DrugBank database was performed against the SFTSV L protein. Molecular dynamics (MD) simulation was performed to investigate the stability of ligand–receptor complexes. Binding free energies in terms of molecular mechanics Poisson-Boltzmann surface area (MMPBSA) were calculated too. The present work outlines the findings about four promising hits—bromfenac, cinchophen, elliptinium, and zaltoprofen—featuring possible binding to the SFTSV L protein.

## Methods

### Protein data preparation

This step is an important part of the in silico drug design approach. The SFTSV L protein’s structure was retrieved from the Protein Data Bank (PDB; https://www.rcsb.org/search) (PDB ID: 6XYA). The protein crystal structure contains bound co-crystalline water molecules that were removed by using the PyMol software^10^⁠. This co-crystalline protein structure contains a bound sodium ion (Na^+^), which was also removed. The protein structure was bound with co-crystalline ligand MGP which was extracted, and its data were saved separately. Then, the co-crystalline ligand, water, and ion-free protein structure were saved in a pdb file and imported into software called AutoDock tools^11^⁠. After that, polar hydrogen and Kollman charges were added. The uniform distribution of charges was taken into account, and the protein coordinates were saved in pdbqt file format.

### Small-molecule database preparation

The DrugBank (https://go.drugbank.com/) database (which is a chemical library) was used for the present work. Two-dimensional (2D) chemical structures of molecules were retrieved from DrugBank in SDF file format. The Open Babel software^12^⁠ was employed to convert all 2D chemical structures into 3D structures, followed by energy minimization and structure optimization. An in-house bash script was used to run the Open Babel software. For structure optimization, an MMFF94 force field built into Open Babel was utilized. Using the steepest descent algorithm^13^⁠, each ligand structure was minimized for 10,000 steps. Then, each minimized structure was saved in the pdbqt file format.

### Molecular-docking–guided virtual screening

For this purpose, the AutoDock Vina software^14^⁠ was used. An in-house bash script was executed to implement the molecular-docking–based virtual screening process. A confined co-crystalline ligand-binding site was regarded as a receptor grid. By means of receptor grid X, Y, and Z coordinates of 2.809, 0.512, and 16.668, respectively, and 20 Å grid box dimensions, an AutoDock Vina configuration file was prepared with an exhaustiveness setting of 8. After successful execution of the docking-based virtual screening, 10 conformers were retained for each ligand. The PyMol software was employed for visual inspection of the docking results. The Maestro-v12.3 visualization ware (Schrödinger Release 2020–1; Maestro, Schrödinger, LLC, New York, NY, 2021) was used for rendering images.

### Docking validation

This validation is a crucial step in molecular-docking–based virtual screening^15^. The 3D structure of the co-crystalline ligand MGP was retrieved from the DrugBank database (https://go.drugbank.com/) (DrugBank ID: DB02716) in the SDF format. By the process described in above subsection, this molecule was prepared and saved in the pdbqt format. Next, the prepared molecule DB02716 was docked with the target protein by means of X, Y, and Z grid coordinates of 2.809, 0.512, and 16.668, respectively, and a grid box dimension setting of 20 Å. Ten docked conformations were recorded. Each docked conformation was superimposed upon its native pose (co-crystalline MGP pose), and root mean square deviation (RMSD) was calculated. For superimposition, the RMSD calculation pair_fit plugin script for the PyMol software (https://pymolwiki.org/index.php/Pair_fit) was used.

### MD simulations

These simulations were conducted by means of GPU-accelerated Gromacs 2018.1^16^⁠ software. The Charmm36^17^⁠ force field was applied to prepare the protein topology. The SwissParam online server-based software^18^⁠ was used to generate the ligand parameterization topology. Each system was solvated via the TIP3P^19^⁠ water model resulting in a 10 × 10 × 10 Å cubic box. An adequate amount (0.15 M) of Na + and Cl‒ ions was added to neutralize each solvated protein–ligand system. The steepest descent algorithm was executed to minimize each system with a maximum of 100,000 steps, and the force was set to the value lesser than 10.0 kJ/mole. Two-stage equilibration steps were analyzed. In the 1st step, i.e., the NVT ensemble step, volume, temperature, and the number of particles were kept constant and maintained for 2 ns. The 2nd step is the NPT ensemble step with constant pressure along with temperature, and the numbers of particles were equilibrated for 10 ns. For each equilibration step, a 100 ns positional restraint was applied to Cα atoms. To maintain a solvent equilibrium, free movements were allowed for the solvent molecules. The linear constraint solver algorithm^20^⁠ was executed to constrain covalent bonds of the system. The particle mesh Ewald^21^⁠ method was applied to long-range electrostatic interactions with a cutoff of 1.2 nm and a Fourier spacing of 1.2 nm. The V-rescale weak coupling technique was used to regulate the temperature (300.00 K) of the system. The Parrinello − Rahman method^22^⁠ was utilized to regulate 1 atm pressure, density, and total energy of the system. Each equilibrated system with acceptable geometry, solPyMOL > pair_fit 6xya_entry_00001_conf_02, ccl-min, and Executive RMS: RMS = 1.301 (33 to 33 atoms) vent orientation was subjected to a 100 ns production run without application of any restraint, followed by a 2 femtosecond (fs) step. The structural coordinates were recorded at 2 picosecond (ps) intervals. After successful completion of an MDS, water and ions were stripped out, followed by Periodic boundary correction (PBC) to refine the trajectories. From the refined trajectories, various parameters, such as RMSD^23^, ⁠root mean square fluctuation (RMSF)^24^, ⁠radius of gyration (Rg)^25^, solvent-accessible surface area (SASA)^26^⁠, and the number of hydrogen bonds (H-bonds) between the ligand and protein were calculated. The VMD software^27^⁠ was utilized to visualize the trajectory and render the images, and the Grace software (https://plasma-gate.weizmann.ac.il/Grace) to plot the data.

### Binding-Energy Calculation

The ligand–protein binding interaction was quantitatively estimated by the widely accepted MMPBSA^28^⁠ approach. The MMPBSA method for the GROMACS (g_mmpbsa) script program^29^⁠ was used to perform the MMPBSA-based binding-energy calculation. To explain the basic working principle of this program, a few known equations are given below. It is a well-known fact that for stability of a protein–ligand complex, the energy of the system must be less than the energy of its individual components. Suppose the term ΔGbind represents the binding free energy of a protein with a ligand. Then, ΔGbind can be expressed as

1 $$\Delta {\text{Gbind }} = {\text{ Gcom }}{-} \, \left( {{\text{GP }} + {\text{ GL}}} \right)$$

where Gcom denotes the free energy of the protein–ligand complex. Terms GP and GL represent the free energy of the unbound protein and ligand in a solvent, respectively. Now, the individual components GP and GL can be expressed in Eqs. (2a) and (2b), respectively.

2a $${\text{GP }} = \, \left( {{\text{EMMP}}} \right) - {\text{TS }} + \, \left( {{\text{Gsolv}}} \right)$$

2b $${\text{GL}} = \, \left( {{\text{EMMP}}} \right) \, - {\text{ TS }} + \, \left( {{\text{Gsolv}}} \right)$$

where T and S denote the temperature and entropy, respectively. The term EMMP represents molecular mechanics potential energy in vacuum, and Gsolv is the free energy of solvation. EMMP can be calculated from the molecular mechanics force field parameters via the formula

3 $${\text{EMMP }} = {\text{ EB }} + {\text{ ENB}}$$

where EB represents bonded interactions, angle dihedrals, and other parameters, and ENB represents nonbonded interactions such as electrostatic (EETS) and van der Waals (EVDW) interactions, respectively. Therefore, Eq. (3) can be written as

4 $${\text{EMMP }} = {\text{ EB }} + \, \left( {{\text{EETS }} + {\text{ EVDW}}} \right)$$

The term ENB can be expressed as

4a $${\text{ENB }} = {\text{ EETS }} + {\text{ EVDW}}$$

From Eqs. (2a) and (2b), Gsolv can be calculated by means of an implicit solvent model. Therefore, this term can be expressed via the contribution of electrostatic or polar solvation energy (GP) and nonelectrostatic or non-polar solvation energy (GNP) to the solvation free energy. The g_mmpbsa script along with the APBS software, compute the above terms. To perform the above-mentioned calculation, snapshots of the last 10 ns of a MD trajectory were extracted. The extracted snapshots along with the tpr and index file were employed as input for the g_mmpbsa program to compute the binding energy.

## Results and discussion

### Docking validation

The success of the molecular-docking–based virtual screening validation step is crucial for such projects. In the present work, various co-crystalline ligands were docked with the SFTS L protein. The docking program generated 10 conformations or docking poses. Each docking pose was superimposed upon the native pose (co-crystalline orientation) of a ligand, MGP. After that, the resultant RMSD (Supplementary Fig. 1a) was found to be ~ 1.8 Å, and the corresponding docking score was − 6.8 kcal/mol. The co-crystalline ligand MGP in its native orientation showed π–π stacking interactions with Phe1703 and Tyr1719; H-bonding interactions with Gln1707, Asp1771, and Leu1772; and hydrophobic interactions with residues Pro1706, Ile1738, and Ile1774 of the SFTS L protein (Supplementary Fig. 1b). Protein–ligand interaction analysis of the docking results revealed that the ligand MGP engages in π–π stacking interactions with Phe1703 and Tyr1719 and H-bonding interactions with Phe1703, Gln1707, Asp1771, and Trp1725. Additionally, hydrophobic interactions with Pro1706, Ile1738, Ile1774, Leu1768, and Leu1772 were found (Supplementary Fig. 1c). The RMSD and interaction analysis strongly supported the validity of the docking protocol^30^⁠. Moreover, based upon the above observations, a strategy was chosen for virtual-screening–based hit identification vis-à-vis the SFTS L protein. Any small-molecule drug that manifested a Vina docking score of more than −7.0 kcal/mol and interactions with Phe1703, Tyr1719, Gln1707, Asp1771, Pro 1706, Ile1738, and Ile1774 was shortlisted and included in a rank. The π–π-type interaction was assumed to be one of the important interactions for ligand binding. The above hypothesis was tested to compile a short list of small-molecules from the DrugBank database.

### Molecular-docking–based virtual screening

In the present study, Autodock Vina was employed as docking software. An in-house bash script was used to execute the docking program to screen out many molecules. Initially, the top 10 molecules were chosen (Table 1) based on a highly negative docking score.

**Table 1:** Docking Score of the Top 10 Ligands.

Short-list rank	Ligand name	Average docking score (kcal/mol)
1	Bromfenac	−8.70
2	Elliptinium	−8.50
3	Oxcarbazepine	−8.20
4	Cinchophen	−8.20
5	Zaltoprofen	−8.00
6	Cyproheptadine	−7.90
7	Epinastine	−7.90
8	Mianserin	−7.80
9	Midazolam	−7.80
10	Phenytoin	−7.70

**Figure 1:**
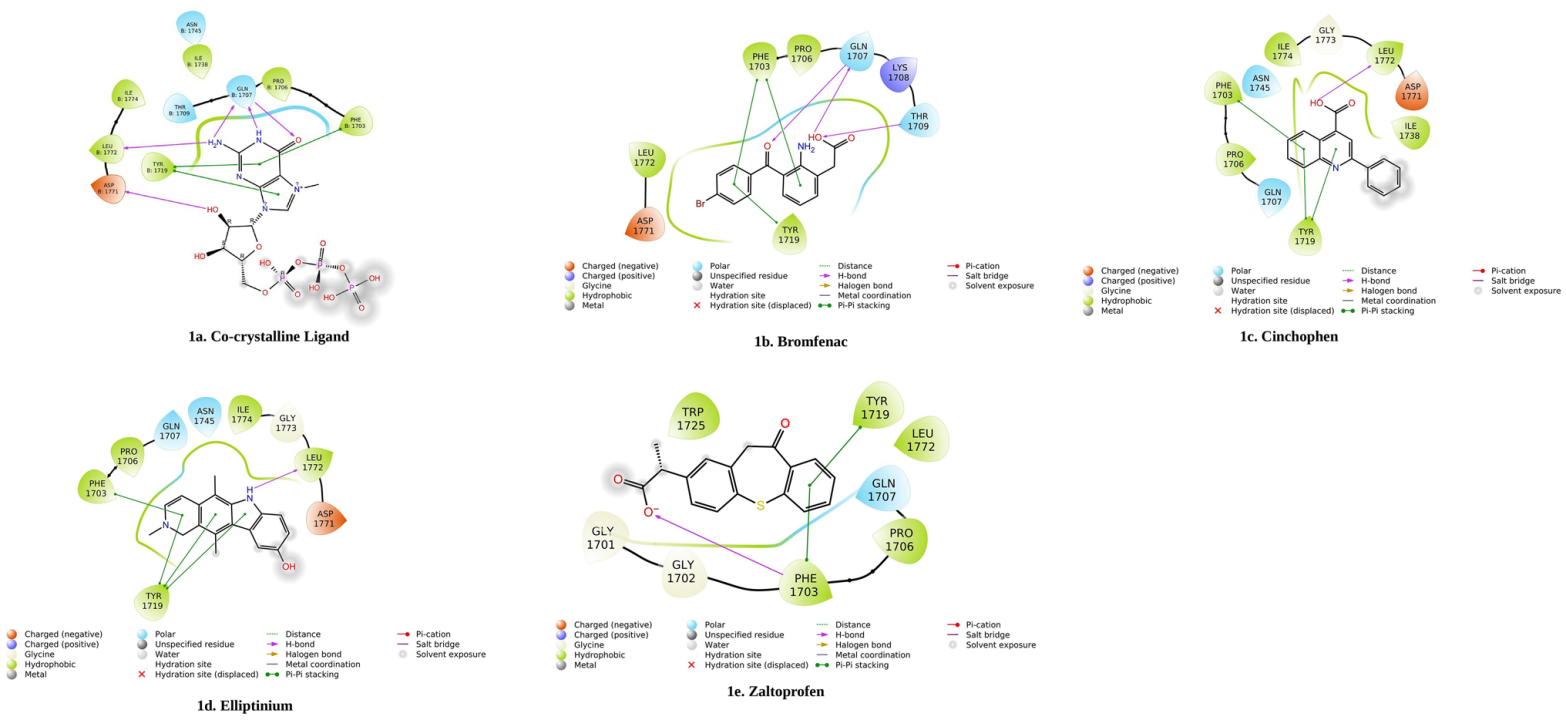
Two-dimensional interactions of top four ligands with the SFTS L protein.

The top ligands, i.e., bromfenac, elliptinium, cinchophen, and zaltoprofen, had Vina docking scores of −8.7, −8.5, −8.2, and −8.0 kcal/mol respectively. Ligand oxcarbazepine showed a docking score −8.20 kcal/mol, but clinically, it is used as an anticonvulsant agent. Therefore, this molecule was not considered further in the present study. Other ligands such as cyproheptadine, epinastine, mianserin, midazolam, and phenytoin had docking scores greater than −8.0 kcal/mol. Bromfenac featured the lowest docking score among all the ligands subjected to the virtual screening (Table 1). According to the interactions with relevant amino acid residues of the SFTS L protein (Fig. 1), the best four molecules were selected. Figure 1a shows the interaction of the co-crystalline ligand with the SFTS L protein. The ligand bromfenac shows π–π interactions with Phe1703 and Tyr1719, an H-bonding interaction with Gln1707, and hydrophobic interactions with Pro1706, Asp1771, and Leu1772 of this protein (Fig. 1b). The ligand cinchophen engages in π–π interactions with Tyr1719 and Phe1703, H-bonding interactions with Leu1772, and hydrophobic interactions with Asp1771, Ile1774, Ile1738, and Pro1706 of the SFTS L protein (Fig. 1c). The ligand elliptinium shows π–π interactions with Tyr1719 and Phe1703, an H-bonding interaction with Leu1772, and hydrophobic interactions with Ile1774 and Pro1706 (Fig. 1d). The ligand zaltoprofen engages in π–π interactions with Phe1703 and Tyr1719, an H-bonding interaction with Phe1703, and hydrophobic interactions with Leu1772, Ile1774, and Pro1706 residues (Fig. 1e).

In the docking analysis, it was found that the ligands bromfenac, cinchophen, elliptinium, and zaltoprofen were engaged in 2 or more π–π interactions. For this reason, these four ligand–protein systems were analyzed further, by MD simulations. Because these are clinically established small-molecule drugs, no theoretical absorption, distribution, metabolism, and excretion analyses were performed on them here.

### MD simulations

MD simulation is widely accepted by the scientific community as a way to assess the stability of a protein–ligand complex. A 100 ns atomistic MD simulation was performed to explore the dynamic properties of each promising protein–ligand complex and to compare them with the dynamic behavior of the ligand-free protein or Apo protein. The average values of every parameter calculated from MD trajectories are presented in Table 2.

**Table 2:** Various Characteristics of the L Protein and Ligands.

Name	Max RMSD-BB* (Å)	Min RMSD-BB (Å)	Avg** RMSD-BB (Å)	Max RMSF (Å)	Min RMSF (Å)	Avg RMSF (Å)	Avg SASA (Å^2^)	Avg Rg (Å)	Avg No. of H-bonds	Avg ∆G_bind_
Apo protein	3.05	0.03	1.63	3.91	0.39	0.89	680.95	13.97	–	–
MGP	2.64	0.4	1.10	4.92	0.36	0.83	673.52	13.83	1.3	−111.42 ± 3.7
Bromfenac	2.92	0.02	1.55	3.80	0.42	0.90	679.19	13.97	0.67	−183.478 ± 0.24
Cinchophen	2.53	0.01	1.24	3.54	0.38	0.83	669.86	13.89	1.56	−145.806 ± 1.3
Elliptinium	2.87	0.02	1.73	2.95	0.37	0.79	659.23	13.86	0.12	−101.738 ± 7.8
Zaltoprofen	2.10	0.01	1.04	2.94	0.35	0.77	675.99	13.91	1.39	−220.095 ± 0.33

*BB: backbone atoms, **Avg: average.

**Figure 2:**
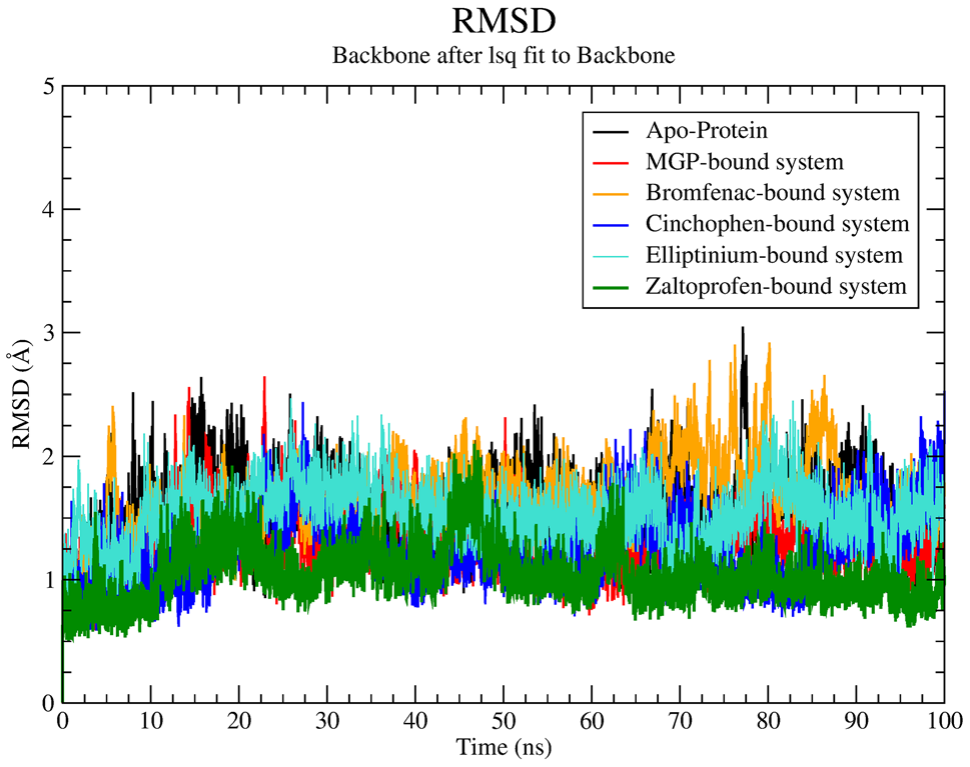
The root mean square deviation (RMSD)-versus-time plot.

RMSD values of each frame were calculated from the total 100 ns MD trajectory and plotted against time (Fig. 2). The RMSD parameter calculated from the MD trajectory denotes the probable changes that transpire in the geometric orientation of a protein structure. RMSD less than 3 Å indicates superior stability of a globular protein^23,31^.⁠ From the average RMSDs in Table 2, it can be concluded that each ligand oscillates at lower RMSD in comparison with the Apo protein (RMSD ~ 1.63 Å) except for the elliptinium–protein system.

Among the 4 protein–ligand systems, the bromfenac–protein system has the highest maximum RMSD. The zaltoprofen–protein system has the lowest RMSD profile among the four systems. Each system oscillates at an RMSD of less than 3.0 Å. An in-depth analysis of Fig. 2 indicates that none of the identified candidate systems undergo abrupt changes in the RMSD profile.

**Figure 3:**
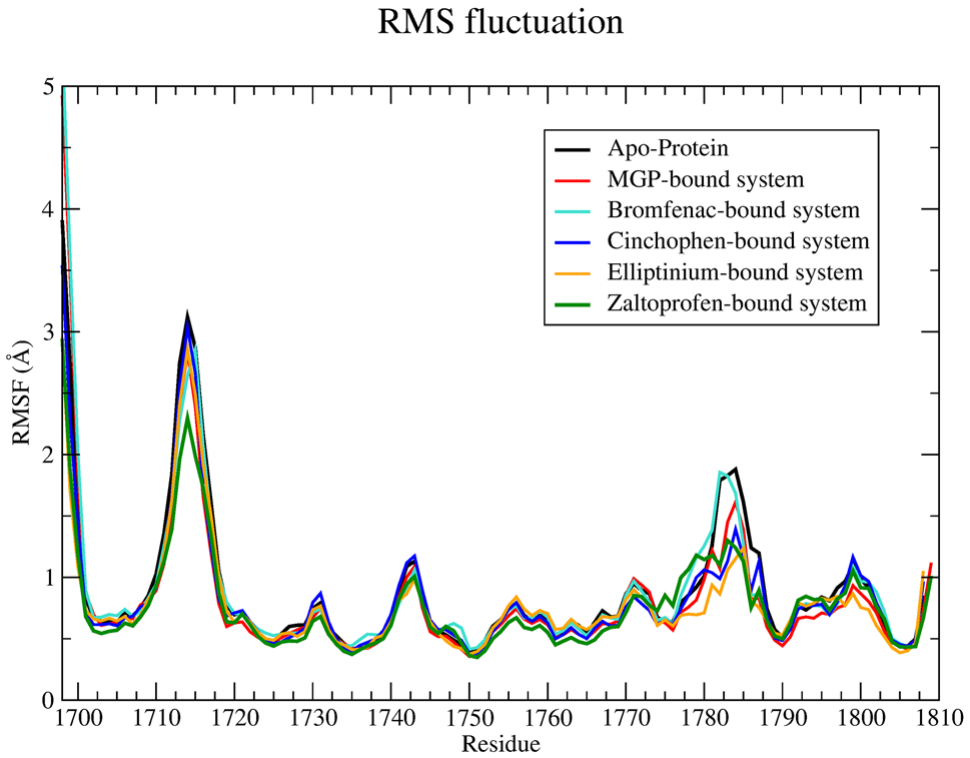
Root mean square fluctuation (RMSF) profiles of each system.

To understand the behavior of individual amino acid residues during the 100 ns MD simulation, RMSF properties were calculated next, and the average values can be found in Table 2. Each system other than the bromfenac system possesses a lower average RMSF than the Apo protein’s RMSF. The bromfenac–protein system has a 0.01 Å higher average RMSF profile than the Apo protein. Considering that MD is a stochastic process, this difference is acceptable^32,33^⁠. For detailed examination of the RMSF profile, RMSF of each amino acid residue was calculated from the 100 ns MD trajectory and plotted against the residue number (Fig. 3). Phe1703, Pro1706, Gln1707, Thr1709, Tyr1719, Asp1771, and Leu1772 are the crucial amino acid residues in the ligand-binding site of the SFTS L protein, and in-depth analysis of Fig. 3 revealed that no significant changes occur when one of the 4 ligands binds to this protein.

**Figure 4:**
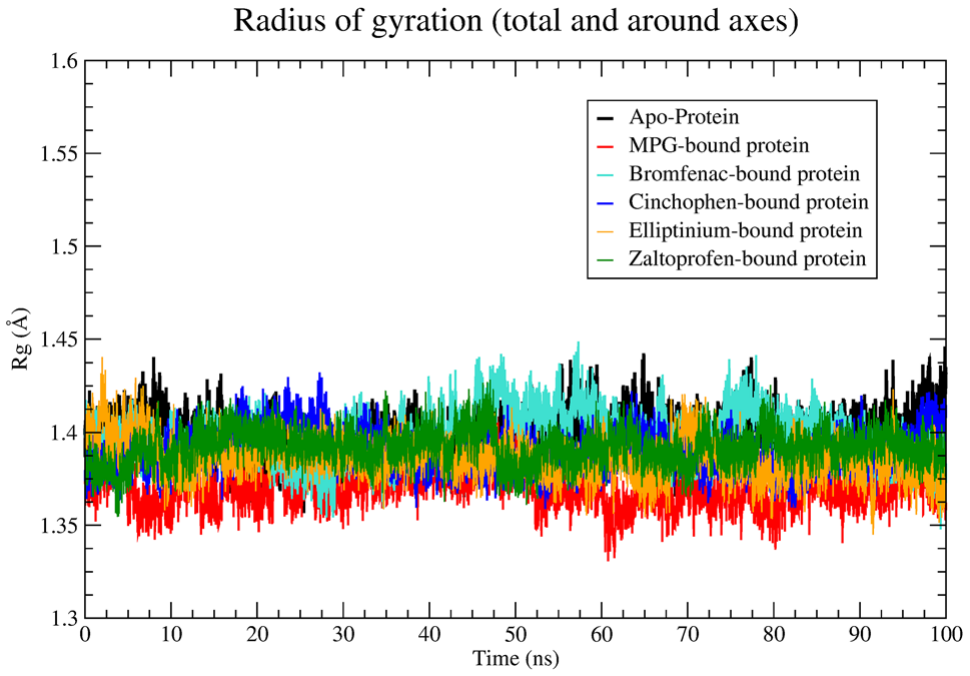
The radius of gyration (Rg)-versus-time plot.

The radius of gyration (Rg) calculated from the MD trajectory indicates the compactness or rigidity of a protein system during the simulation^25^⁠. A consistent Rg profile indicates that the protein system may be stable and does not undergo any significant structural changes or distortions during the simulation. Average values of Rg calculated for each system can be found in Table 2. To understand changes of Rg with time, a plot was constructed (Fig. 4).

In Table 2, readers can see that each system has the average Rg either similar to or less than the Apo protein’s Rg of ~ 13.97 Å. Analysis of the Rg-versus-time plot constructed for each system indicates that there are no significant changes in the Rg profile. In accordance with the above observations, it can be stated that in each system, the SFTS L protein retains its geometric orientation and forms a stable complex with each of the identified promising ligands.

**Figure 5:**
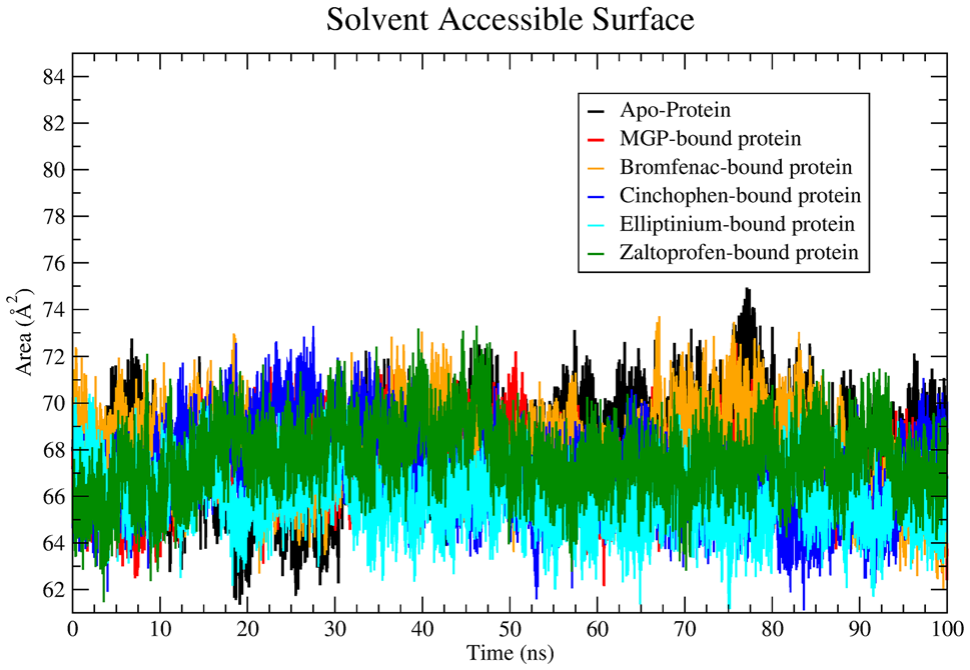
The solvent-accessible surface area (SASA)-versus-time plot.

SASA parameter calculated from the MD trajectory provides insight into the events in the protein–ligand contact. If, ligand binding to a protein is considered as a solvent replacement approach, then lower SASA value means that the protein or ligand is less exposed to the solvent. That is, the ligand is located deep inside the binding pocket^26^⁠. According to Table 2, SASA values of each ligand–protein system—as calculated from the 100 ns atomistic MD trajectory—are less than SASA of the ligand-free protein. This critical observation strongly indicates that during the 100 ns MD simulation, each ligand is situated inside the receptor-binding pocket. To understand the changes of SASA with time, a plot was constructed (Fig. 5).

The binding energy in terms of MMPBSA was also calculated from the 100 ns MD trajectory. Highly negative binding energy implies stable binding of a ligand to a protein^15^⁠. The average values of the binding energy in kJ/mol are shown in Table 2. The binding energy of co-crystalline ligand MGP was calculated from molecular dynamics trajectory and compared with the binding energy of identified binders. The zaltoprofen–protein system has a highly negative binding energy −220.095 kJ/mol. The elliptinium–protein system features a moderate binding energy −101.738 kJ/mol. The bromfenac and cinchophen systems show binding energies of −183.478 and −145.806 kJ/mol, respectively. In addition, from the Table 2 it can be observed that the binding energy of each identified hits (except Elliptinium) were found to be high negative in compare to MGP bound system. The MMPBSA-based binding-energy profile of the identified ligands toward the SFTS L protein suggests that each ligand binds stably. This statement is further supported by data on other parameters, such as RMSD, RMSF, Rg, and SASA, calculated from the MD trajectory.

In conclusion, we report the identification of small-molecules (from the DrugBank database) that may stably bind to the SFTS L protein. The Autodock Vina docking software was used to implement the virtual-screening process. By the molecular-docking–based virtual screening, 10 small-molecule hits were identified on the basis of the binding energy and protein–ligand interactions. The shortlisted molecules are established drugs in clinical practice. Therefore, no theoretical absorption, distribution, metabolism, and excretion analyses were conducted here. MD simulations were performed on the top 4 ligands with docking-based binding energy lesser than −8.0 kcal/mol toward the SFTS L protein. All the molecules subjected to the MD simulations engage in π–π interactions with amino acid residues Phe1703 and Tyr1719 of the protein. Various parameters, e.g., RMSD, RMSF, RoG, and SASA, were calculated. Moreover, binding affinity of the ligands for the SFTS L protein was computed from a 100 ns MD trajectory. Analysis of each MD simulation parameter and the binding-energy profile strongly indicate stable binding of each of the 4 ligands to the SFTS L protein. Zaltoprofen manifested the best binding energy −220.095 kJ/mol.

In conclusion, this study presents four clinically used small-molecules exhibiting stable binding with SFTS L protein for up to 100 ns of MD simulation time. Nonetheless, proper biological evaluation is necessary to determine their efficacy against SFTS.

## Supplementary Information

Supplementary Information.

## Data Availability

All data will be available upon request to the corresponding author.
