# Supplementary material for: Potential efficacy of existing drug molecules against severe fever with thrombocytopenia syndrome virus: an in silico study
Source: Sci Rep. 2021 Oct 21;11:20857. doi: 10.1038/s41598-021-00294-7 (PMC8531283; doi:10.1038/s41598-021-00294-7)
Supplement: Supplementary file 1 — Supplementary Information. [file 41598_2021_294_MOESM1_ESM.docx]

**Potential Efficacy of Existing Drug Molecules against Severe Fever with Thrombocytopenia Syndrome Virus: An *in Silico* Study**

**Supplementary Figure 1a: Green color ligand= Native pose, Yellow color ligand=Docking conformation. RMSD=1.8Å**


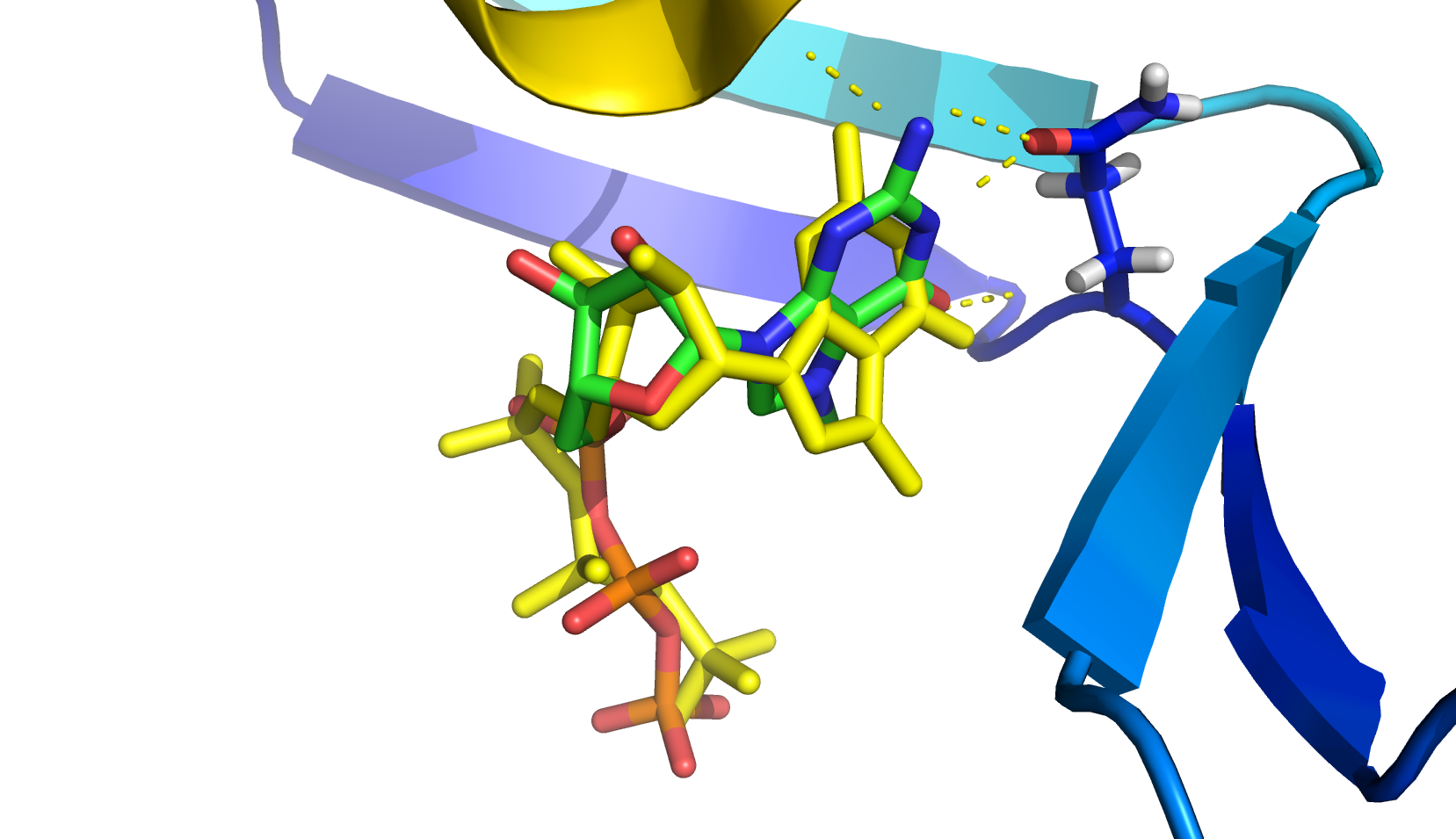


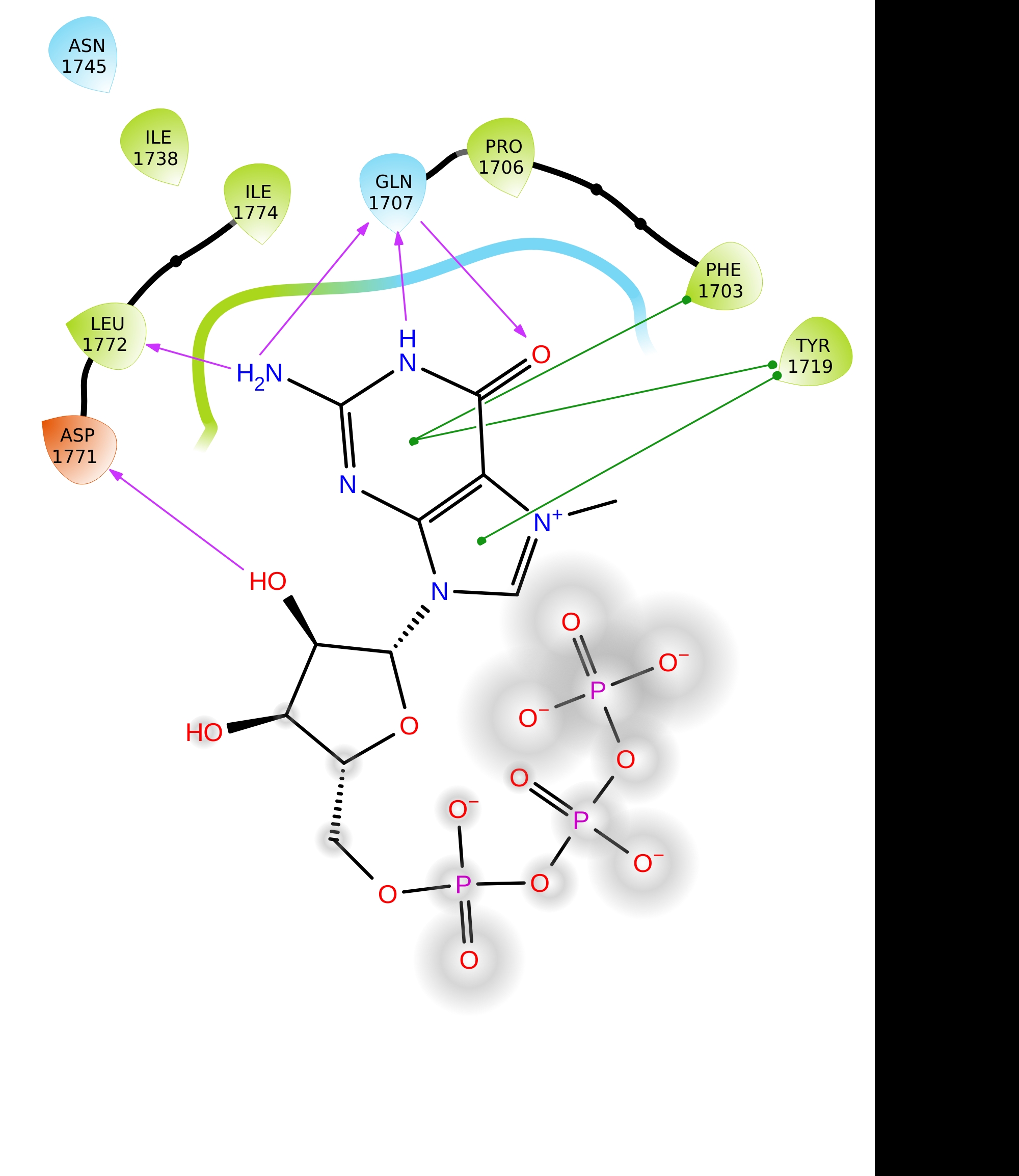


**Supplementary Figure 1b**


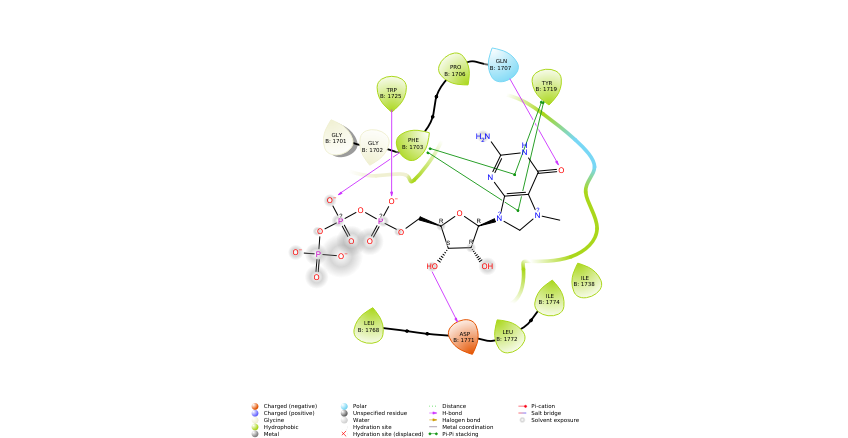


**Supplementary Figure 1c**
